# Indirect regeneration in *Ficus lyrata* Warb. and metabolite profiles influenced by nitric oxide and Plant growth regulators

**DOI:** 10.1186/s12870-023-04339-z

**Published:** 2023-06-17

**Authors:** Ruhollah Abdolinejad, Mohamadreza Salehi Salmi

**Affiliations:** 1 Department of Horticultural Science, College of Agriculture, Shiraz University, Box 65186-71441, Shiraz, Iran; 2 Department of Horticultural Science, Agricultural Sciences and Natural Resources University of Khuzestan, Mollasani, IR 6341773637 Khuzestan Iran

**Keywords:** Indirect morphogenesis, Fiddle leaf fig tree, Nitric oxide, Primary metabolites, Free amino acids

## Abstract

**Background:**

To establish an indirect regeneration protocol in *Ficus lyrata*, a three-phase experiment (callus induction, morphogenic callus induction, and plant regeneration) based on auxin, cytokinin, and nitric oxide interactions was designed and implemented using leaf explants. The metabolite profiles (amino acid profile, total phenolic content, total soluble sugars, and total antioxidant activity) alteration patterns were also investigated to determine the metabolites contributing to the progress of each phase.

**Results:**

Results demonstrated that 11 out of 48 implemented treatments resulted in morphogenic callus induction (morphogenic treatments), and nitric oxide played a key role in increasing efficiency from 13 to 100%. More importantly, nitric oxide cross-talk with cytokinins was necessary for shoot regeneration from morphogenic calli. Only 4 out of all 48 implemented treatments were capable of shoot regeneration (regenerative treatments), and among them, PR42 treatment led to the highest shoot regeneration rate (86%) and maximum mean number of shoot/explant (10.46). Metabolite analyses revealed that the morphogenic and regenerative treatments followed similar metabolite alterations, which were associated with increased biosynthesis of arginine, lysine, methionine, asparagine, glutamine, histidine, threonine, leucine, glycine, serine amino acids, total soluble sugars content, and total antioxidant activity. On the contrary, non-morphogenic and non-regenerative treatments caused the accumulation of a significantly greater total phenolic content and malondialdehyde in the explant cells, which reflexed the stressful condition of the explants.

**Conclusions:**

It could be concluded that the proper interactions of auxin, cytokinins, and nitric oxide could result in metabolite biosynthesis alterations, leading to triggering cell proliferation, morphogenic center formation, and shoot regeneration.

**Supplementary Information:**

The online version contains supplementary material available at 10.1186/s12870-023-04339-z.

## Background

Fiddle-leaf fig (*Ficus lyrata* Warb.) is an evergreen broad-leaved tree belonging to the Moraceae family and originated from tropical forests of western and central Africa [1]. *F. lyrata* is a decorative ornamental plant due to its large, deep green waxy, and violin-shaped leaves [2], which is widely used as a popular landscape plant in tropical and subtropical areas or a foliage interiorscaping plant in temperate zones. The global market demands [3] for this ornamental species can be met by establishing successful de novo regeneration protocols. However, the *Ficus* species, including *F. lyrata*, are known in vitro recalcitrant woody species, especially where indirect regeneration is the purpose [4].

Nowadays, genetic engineering technology including transgenesis and genome editing are novel technologies to create new elite genotypes with improved ornamental features [5]. However, the prerequisite for taking advantage of these technologies is establishing a reliable indirect regeneration protocol for every plant genotype, considered the main obstacle and bottleneck for breeding programs of many recalcitrant woody species [6, 7]. Moreover, establishing indirect regeneration systems in different plant species could also be exploited for various breeding programs such as protoplast fusion, mutation breeding, synthetic seed production, ploidy manipulation, in vitro selections, secondary metabolite production, etc., [4].

However, plant species are widely different in competency for de novo regeneration [8], ranging from easy-to-propagate to woody recalcitrant species. Recalcitrant species display difficulties in morphogenesis under in vitro conditions, requiring broad-spectrum manipulation in the culture medium and environmental conditions during the different phases i.e., callogenesis, morphogenesis, and shoot regeneration. Plant media supplementary additives such as PGRs, some vitamins, amino acids, antioxidants, etc., alone or in combination could effectively influence the de novo regeneration process. Numerous studies in various plant species highlighted the auxin/cytokinin interaction as the main factor in promoting plant de novo direct and indirect regeneration [9]. However, optimizing an appropriate auxin/cytokinin ratio and using supplementary additives in the medium is a common and necessary approach to stimulate recalcitrants to induce morphogenic calli [4, 7, 9, 10]. In the case of the *Ficus* genus, it has been well documented that an interaction between a strong cytokinin like TDZ and an auxin like IBA or NAA could lead to the formation of morphogenic calli, meristematic centers, and even direct shoot development in different explants [3, 4, 11–13].

Sodium nitroprusside (SNP), a nitric oxide (NO) donor has been highly recommended as an effective additive in culture medium to enhance de novo regeneration efficiency in different plant species [14, 15]. The cross-talk of NO as a multifunctional signaling molecule [16] with auxin and cytokinin (CK) could be considered a promising treatment to overcome in vitro recalcitrancy, especially in woody species [17]. Ötvös [15] revealed that a synergistic interaction between NO and auxin has significantly stimulated cell division and somatic embryogenesis in protoplast-derived cells of alfalfa. The hypothesis was proved when NO synthesis inhibitor or NO scavenger suppressed both cell division and somatic embryogenesis. The stimulatory effect of NO on callogenesis, somatic embryogenesis, shoot proliferation, and rooting of explants has also been frequently emphasized in numerous studies, although, the precise involved molecular mechanisms have remained unclear [17].

To better understand the indirect regeneration process in each phase, performing a comparative investigation on metabolite alteration trends is very important. Cell metabolite's alteration patterns give essential information to distinguish key metabolites that are well-linked to developing de novo organogenesis stages. Then as metabolite markers, they could be used exogenously in commercial or scientific projects.

The current study was designed and carried out to establish a high-efficiency indirect regeneration protocol in *F. lyrata* using leaf explants for large-scale micropropagation and its other biotechnological applications. In particular, the response of the leaf explants to PGRs × NO interactions and the metabolite profiles at the different regeneration stages were investigated.

## Results

**Fig. 1:**
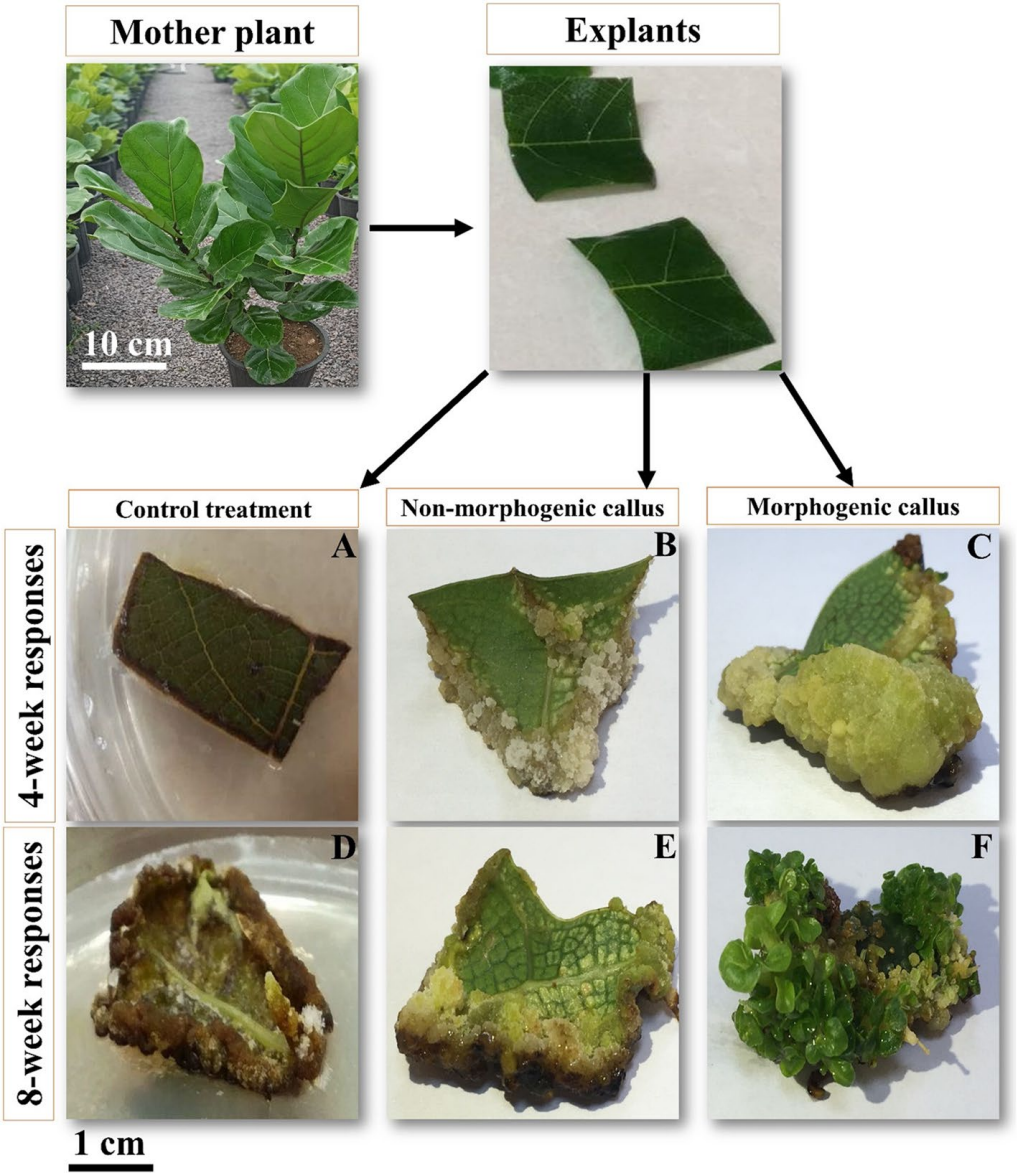
Callogenesis and morphogenesis responses in leaf explants of *F. lyrata* cultured on MT medium and different auxin × cytokinin × nitric oxide interactions. **A** and **D** display the explant cultured on the control treatment (CI0 treatment) characterized by no capability of callogenesis in the 4^th^ week and death of the explants in the 8^th^ week. **B** and **E** represent the explants of a non-morphogenic treatment (CI46 treatment) representing the formation of non-organogenic calli in the 4^th^ week and then the browning calli in the 8^th^ week. **C** and **F** represent explants of morphogenic treatment (CI33 treatment) characterized by pluripotent calli induction in the 4^th^ week and then the formation of morphogenic centers in the 8^th^ week

To establish a de novo indirect regeneration system, a three-phase experiment including callus induction (CI phase), morphogenic callus induction (MCI phase), and plant regeneration (PR phase), responses for the interactions between different PGRs and NO concentrations was designed as displayed in Tables 1 and 2, and Supplementary data, S3. The typical morphometric responses scored at the end of each experiment are shown by the responses of the explants as browning, non-morphogenic callus, and morphogenic callus used to score the % responses at each phase (Fig. 1).

**Table 1:** Effect of different auxin ✕ cytokinin ✕ nitric oxide interactions on the browning index, callus induction (%), and morphogenic callus induction (%) parameters of *F. lyrata* leaf explants cultured on MT medium

Nitric oxide (μM)	TDZ (μM)	IBA (μM)	Treatment code	Browning (index)	Callus induction (%)	Morphogenic callus induction (%)
0	0	0	CI0	5 ± 0 a	0 ± 0 l	0 ± 0 g
		2.4	CI1	5 ± 0 a	0 ± 0 l	0 ± 0 g
		4.9	CI2	5 ± 0 a	0 ± 0 l	0 ± 0 g
		9.8	CI3	5 ± 0 a	0 ± 0 l	0 ± 0 g
	4.5	0	CI4	3 ± 0 bcd	0 ± 0 l	0 ± 0 g
		2.4	CI5	2.33 ± 0.26 c-f	33.33 ± 4.6 ghi	0 ± 0 g
		4.9	CI6	3.33 ± 0.13 bc	46.66 ± 6.3 fg	13.33 ± 2.6f
		9.8	CI7	2.66 ± 0.23 b-e	66.66 ± 3.2 de	0 ± 0 g
	9	0	CI8	2.33 ± 0.31 c-f	33.33 ± 6.1 ghi	0 ± 0 g
		2.4	CI9	2 ± 0 d-g	66.66 ± 4.6 de	0 ± 0 g
		4.9	CI10	2.66 ± 0.33 b-e	86.66 ± 6.8 abc	13.33 ± 3.13 f
		9.8	CI11	3 ± 0 bcd	66.66 ± 5.3 de	0 ± 0 g
10	0	0	CI12	2.33 ± 0.13 c-f	0 ± 0 l	0 ± 0 g
		2.4	CI13	3 ± 0 bcd	0 ± 0 l	0 ± 0 g
		4.9	CI14	2.33 ± 0.23 c-f	13.33 ± 2.6 jkl	0 ± 0 g
		9.8	CI15	2.33 ± 0 c-f	20 ± 3.7 ijk	0 ± 0 g
	4.5	0	CI16	1.33 ± 0.13 f-i	0 ± 0 l	0 ± 0 g
		2.4	CI17	0.66 ± 0.07 hi	93.33 ± 2.3 ab	60 ± 8.23 bc
		4.9	CI18	0.66 ± 0.07 hi	86.66 ± 2.8 abc	46. ± 3.33 66 d
		9.8	CI19	2 ± 0.58 d-g	66.66 ± 6.3 de	13.33 1.31f
	9	0	CI20	2.66 ± 0.76 b-e	0 ± 0 l	0 ± 0 g
		2.4	CI21	1.66 ± 0.13 e–h	53.33 ± 4.6 ef	0 ± 0 g
		4.9	CI22	0.66 ± 0.07 hi	100 ± 0 a	53.33 ± 4.46 cd
		9.8	CI23	1 ± 0.18 ghi	80 ± 4.3 bcd	20 ± 3.31 ef
20	0	0	CI24	2.33 ± 0.33 c-f	0 ± 0 l	0 ± 0 g
		2.4	CI25	1.66 ± 0.33 e–h	6.66 ± 1.3 kl	0 ± 0 g
		4.9	CI26	2.33 ± 0.33 c-f	53.33 ± 6.4 ef	0 ± 0 g
		9.8	CI27	2.66 ± 0.37 b-e	6.66 ± 1.3 kl	0 ± 0 g
	4.5	0	CI28	1.66 ± 0.27 e–h	0 ± 0 l	0 ± 0 g
		2.4	CI29	0.33 ± 0.05 i	100 ± 0 a	66.66 ± 4.46 b
		4.9	CI30	2.33 ± 0.33 c-f	100 ± 0 a	26.66 ± 3.31 e
		9.8	CI31	0.33 ± 0.03 i	73.33 ± 6.8 cd	0 ± 0 g
	9	0	CI32	1.66 ± 0.23 e–h	0 ± 0 l	0 ± 0 g
		2.4	CI33	0.33 ± 0.03 i	100 ± 0 a	100 ± 0 a
		4.9	CI34	1 ± 0.18 ghi	86.66 ± 6.4 abc	53.33 ± 6.66 cd
		9.8	CI35	1.33 ± 0.13 f-i	73.33 ± 4.2 cd	0 ± 0 g
40	0	0	CI36	3.66 ± 0.42 b	0 ± 0 l	0 ± 0 g
		2.4	CI37	2.33 ± 0.33 c-f	33.33 ± 3.5 ghi	0 ± 0 g
		4.9	CI38	2.66 ± 0.33 b-e	53.33 ± 3.3 ef	0 ± 0 g
		9.8	CI39	1.33 ± 0.23 f-i	80 ± 3.8 bcd	0 ± 0 g
	4.5	0	CI40	1 ± 0 ghi	0 ± 0 l	0 ± 0 g
		2.4	CI41	2.33 ± 0.33 c-f	26.66 ± 2.6 hij	0 ± 0 g
		4.9	CI42	3.33 ± 0.33 bc	33.33 ± 2.8 ghi	0 ± 0 g
		9.8	CI43	2.33 ± 0.35 c-f	40 ± 3.3 fgh	0 ± 0 g
	9	0	CI44	1.33 ± 0.13 f-i	0 ± 0 l	0 ± 0 g
		2.4	CI45	2 ± 0.28 d-g	66.66 ± 5.4 de	0 ± 0 g
		4.9	CI46	2.66 ± 0.33 b-e	86.66 ± 4.2 abc	0 ± 0 g
		9.8	CI47	3.66 ± 0.33 b	46.66 ± 4.6 fg	0 ± 0 g

Means in each column with the same letters are not significantly different at *p* < 0.05, according to Duncan’s multiple range tests. Each treatment includes three replicates, and each replicate contains five explants. Each value represents the mean ± SE

**Table 2:** The interactions of different BAP × TDZ × NO concentrations on plant regeneration from morphogenic calli that were evaluated

BAP (μM)	TDZ (μM)	Nitric oxide (μM)	NAA (μM)
0	0	0	0.53
8.9	1.14	10	0.53
17.8	2.25	20	0.53
	4.5	40	0.53

**Fig. 2:**
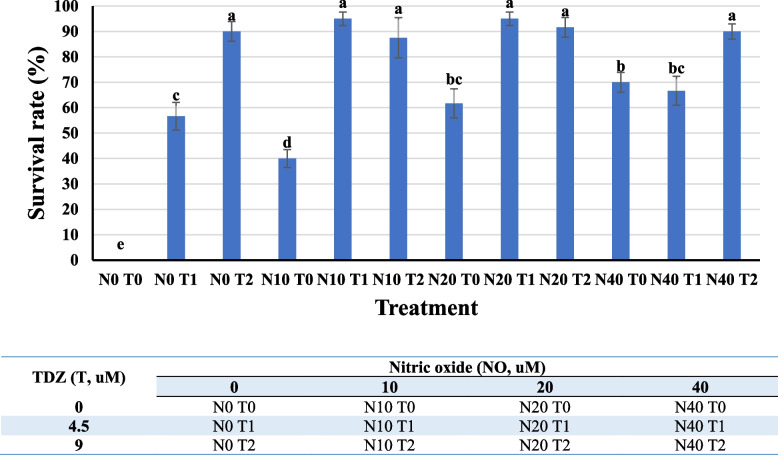
Effect of different nitric oxide × thidiazuron interactions on the percentage survival of leaf explants of *F. lyrata*, four weeks after culturing on callus induction media

Results of the initiation of explants in MT media supplemented with TDZ × NO revealed that the survival (%) of the explants at the end of 4 weeks was affected by this interaction (Fig. 2). A maximum % of 95 surviving explants could be obtained in the N10 T1 (nitric oxide 10 μM + thidiazuron 4.5 μM) medium. Accordingly, as shown in Fig. 2, the highest survival percentage was observed in N0T2, N10T1, N10T2, N20T1, N20T2, and N40T2 treatments that were significantly different from the other treatments whilst the control treatment showed the lowest survival percentage with 0% surviving explant in this treatment (Fig. 2). These explants were then used to subculture into the various morphogenic callus induction media (CI).

### Callus induction and morphogenic callus formation

In the callus induction media all other evaluated parameters, including browning index, callus induction, and morphogenic callus induction, were significantly affected by TDZ × IBA × NO interactions (Supplementary data, Table S1).

The highest browning index was observed in the control (CI0), CI1, CI2, and CI3 treatments, which were significantly different from the other treatments (Table 1 and Fig. 1D, E). Indeed, the explants in the media without either NO or TDZ showed the highest browning index, whilst CI29, CI31, and CI33 treatments showed the lowest browning index when the culture media contained a mid-range concentration of NO.

The results also demonstrated that callus induction occurred in a range of 6.66% to 100% in 33 out of all 48 CI treatments (Table 1). However, treatments with a mid-range concentration of NO, TDZ, and IBA led to the best result, so that, of all treatments, CI29, CI30, and CI33 displayed the maximum callus induction (%) (Table 1 and Fig. 1C). On the contrary, treatments without either TDZ or IBA in the culture media, including CI0, CI1, CI2, CI3, CI4, CI12, CI13, CI16, CI20, CI24, CI28, CI32, CI36, CI40, and CI44, were not capable of callus induction in explants (Table 1 and Fig. 1A).

Results revealed that in the MCI phase, the implemented treatments were divided into two distinct groups namely morphogenic treatments (CI6, CI10, CI17, CI18, CI19, CI22, CI23, CI29, CI30, CI33, and CI34) and non-morphogenic treatments (CI0-CI5, CI7- CI9, CI11-CI16, CI20, CI21, CI24-CI28, CI31, CI32, CI35, and CI36-CI47) based on their capability of morphogenic callus induction. Accordingly, the range of morphogenic callus induction was recorded between 13.33% to 100% in 11 out of all those 33 treatments which had produced calli in the CI phase (Table 1). Among them, the CI33 treatment which contained an interaction of a mid-range concentration of NO (20 μM) with a low concentration of IBA (2.4 μM) and a high concentration of TDZ (9 μM), gave rise to the highest (100%) morphogenic callus induction and was significantly different from the other treatments (Table 1, Fig. 1F). Except for the control treatment (CI0) and non-morphogenic treatments, the lowest morphogenic callus induction (13.33%) was found in CI6, CI10, and CI19 treatments (Table 1, Fig. 1E).

### Plant regeneration

Results demonstrated that the interactions of BAP × TDZ × NO had significantly affected shoot regeneration from morphogenic calli, as well as the parameters of the number of shoots/explant and shoot length (Supplementary data, Table S2). Morphogenic calli placed on a combination of media shown in Table 2 below were studied at the end of four weeks for shoot regeneration responses. Out of the 48 combinations evaluated (Supplementary data, Table S3), the media supplemented with BA (17.8 uM), TDZ (2,25; 4.5 um) and NO (10, 20 um) alone responded. These results are provided in Table 3. NO at 0 uM and 40 uM showed no shoot regeneration from morphogenic calli even though the media were supplemented with BA (17.8 uM) and TDZ (2,25; 4.5 um). NAA (0.53 uM) did not influence the regenerative responses (Supplementary data, Table S3).

**Table 3:** Effective interactions of auxin × cytokinins × nitric oxide on shoot regeneration (%), number of shoots/explant, and shoot length parameters of *F. lyrata* explants cultured on MT medium. All other combinations were ineffective

BAP (μM)	TDZ (μM)	Nitric oxide (μM)	NAA (μM)	Treatment code	Regeneration (%)	Shoot/explant	Shoot length (cm)
17.8	2.25	0	0.53	PR40	0 ± 0 d	0 ± 0 e	0 ± 0 d
		10	0.53	PR41	46.66 ± 4.66 c	3.41 ± 0.93 d	0.86 ± 0.12 c
		20	0.53	PR42	86.66 ± 6.33 a	10.46 ± 2.11 a	1.07 ± 0.13 a
		40	0.53	PR43	0 ± 0 d	0 ± 0 e	0 ± 0 d
	4.5	0	0.53	PR44	0 ± 0 d	0 ± 0 e	0 ± 0 d
		10	0.53	PR45	53.33 ± 8.46 b	4.8 ± 1.33 c	0.94 ± 0.09 b
		20	0.53	PR46	86.66 ± 6.13 a	6.06 ± 1.66 b	1.03 ± 0.14 a
		40	0.53	PR47	0 ± 0 d	0 ± 0 e	0 ± 0 d

Means in each column with the same letters are not significantly different at *p* < 0.05, according to Duncan’s multiple range tests. Each treatment includes three replicates, and each replicate contains five explants. Each value represents the mean ± SE

**Fig. 3:**
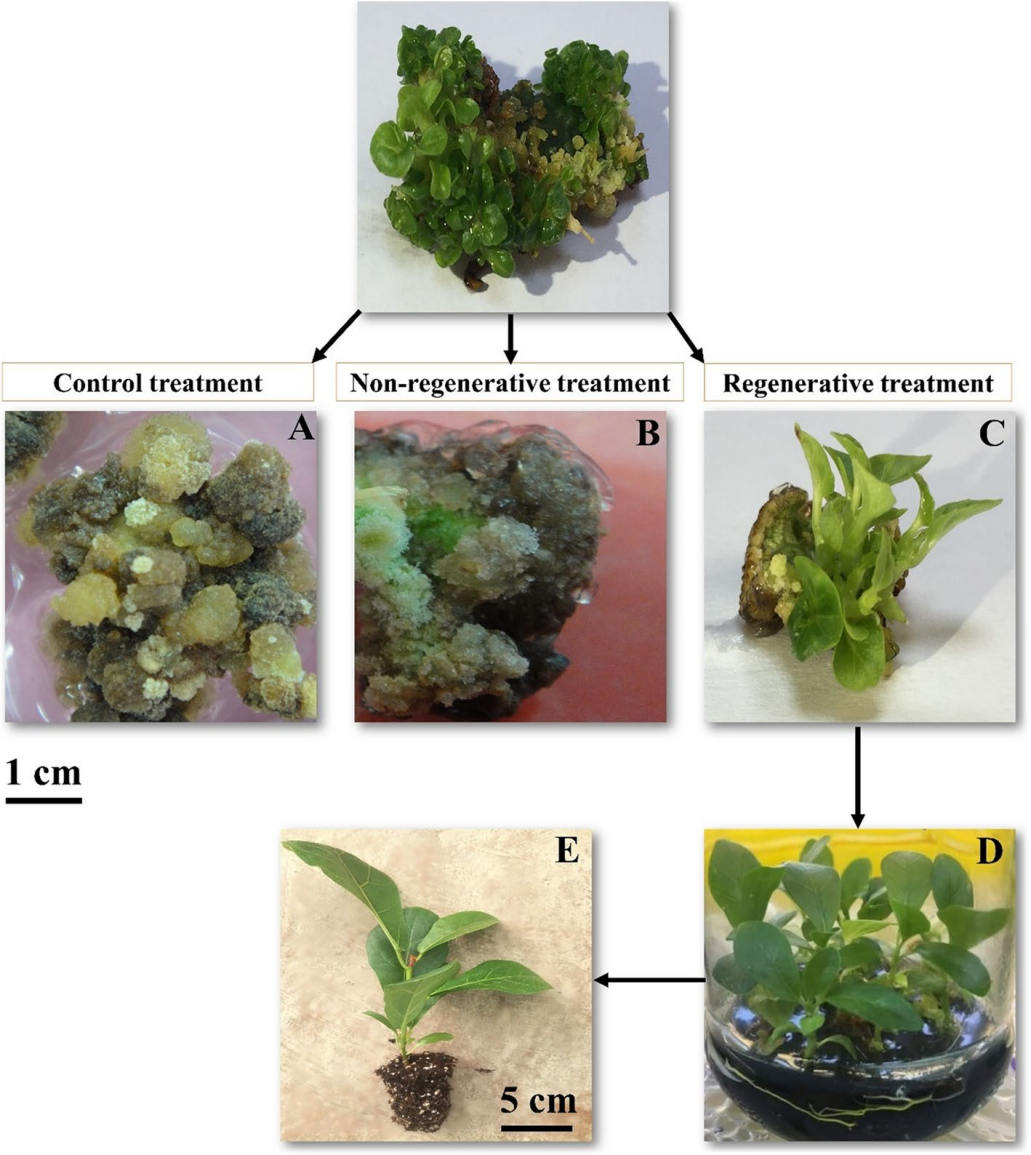
Indirect shoot regeneration from induced morphogenic calli, rooting, and acclimatization of *F. lyrata* plants. **A** represents explants of the control treatment (PR0 treatment), **B** shows an explant belonging to the non-regenerative treatment (PR26 treatment), and **C** represents regenerated shoots from an explant cultured on organogenic treatment (PR42 treatment) at the end of the 4^th^ week. **D** and **E** represent the successful rooting and acclimatization of the regenerated plants on rooting medium and greenhouse conditions, respectively

Like the MCI phase, treatments in the PR phase were divided into two groups based on their capability of shoot regeneration from morphogenic calli. Accordingly, the results of the mean comparisons revealed that only four treatments, namely PR41, PR42, PR45, and PR46, out of all 48 applied treatments could convert the morphogenic calli to shoot, so they were recognized as regenerative treatments, and the other treatments including the control treatment (PR0) which were not capable of regenerating shoot from morphogenic calli were distinguished as non-regenerative treatments (Table 2, Fig. 3A, B). Among the regenerative treatments, PR42 and PR46 treatments with 86.66%, and PR41 treatment with 46.66% shoot regeneration displayed the highest and the lowest regeneration rates, respectively (Table 3). The PR42 treatment also resulted in the highest number of regenerated shoots, with an average number of 10.46 shoot/explant (Table 3, Fig. 3C), and the lowest number of regenerated shoots was recorded in the PR41 treatment, with a mean number of 3.41 shoot/explant. Similarly, a significant difference between treatments was observed in the shoot length parameter, so that the PR42 and PR46 treatments exhibited the highest shoot length with a mean of 1.07 and 1.03 cm, respectively, and the PR41 treatment showed the lowest shoot length with an average of 0.86 cm (Table 3). Rooting and acclimatization of explants were also successfully performed as shown in Fig. 3D, E.

### Metabolite profile

**Fig. 4:**
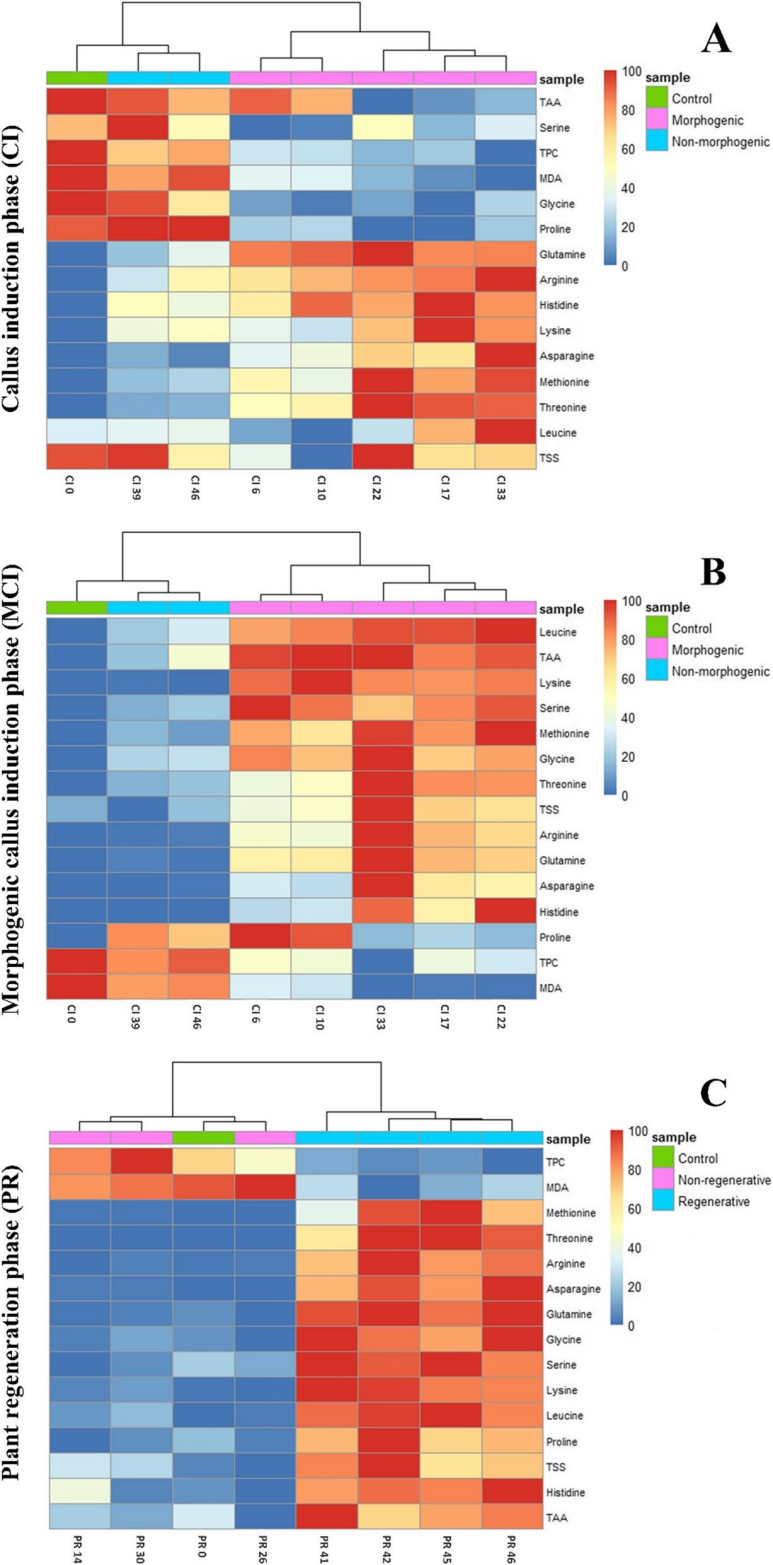
Heatmap of metabolites related to different CI, MCI, and PR phases of *F. lyrata* indirect regeneration. Rows correspond to compounds and columns to the treatment codes. Treatment codes including CI and PR series are defined in Table 1 and Supplementary data, Table S3, respectively

The results of one-way ANOVA indicated that metabolites, including the contents of amino acids, TSS, TPC, MDA, and TAA activity, were significantly affected by different treatments in all three CI, MCI, and PR phases (Supplementary data, Table S4). Indeed, the metabolite alteration trends in every phase were closely related to the interaction and concentration of auxin × CKs × NO and the capability of each treatment to promote callus induction, morphogenic callus formation, and plant regeneration (Fig. 4).

In the CI phase, those treatments that resulted in the highest callus induction and quality (CI33, CI22, CI17) had biosynthesized significantly higher amounts of amino acids like arginine, lysine, methionine, asparagine, glutamine, histidine, threonine, and leucine and conversely, treatments with no capability of callus induction (CI0) or very poorly induced-callus (CI6, CI10, CI39, and CI46) had biosynthesized significantly lower amounts of the above mentioned amino acids, however, they significantly accumulated higher contents of serine and proline amino acids as well as TAA activity and greater TPC, TSS, and MDA contents (Fig. 4 and Supplementary data; Table S4).

The metabolite alteration of the MCI phase also followed the same trend as the CI phase. Indeed, results indicated that the biosynthesis and accumulation of arginine, lysine, methionine, asparagine, glutamine, histidine, threonine, and leucine amino acids showed an increasing trend in the explants of morphogenic treatments (CI33, CI22, and CI17) compared to the control and non-morphogenic treatments (CI0, CI39, and CI46). Furthermore, they also displayed a significant increase in glycine and serine biosynthesis, TSS accumulation, and TAA activity in the MCI stage (Fig. 4 and Supplementary data; Table S4). On the other hand, while the morphogenic treatments (CI33, CI22, and CI17) accumulated negligible contents of TPC, proline, and MDA, the non-morphogenic treatments (CI0, CI39, and CI46) had accumulated significantly high levels of them in their cells (Fig. 4 and Supplementary data; Table S4).

The results also demonstrated that BAP × TDZ × NO × NAA cross-talks significantly affected the metabolite profiles in the PR phase. According to the mean comparison analyses (Supplementary data; Table S4) and as illustrated in the heat map (Fig. 4), significant differences were recognized in biosynthesis and accumulation of metabolites between regenerative (PR41, PR42, PR45, and PR46) and selective non-regenerative (PR0, PR14, PR26, and PR30) treatments. Accordingly, except for TPC and MDA contents, which displayed a decreasing trend, all other metabolites, including the content of arginine, lysine, methionine, asparagine, glutamine, histidine, threonine, leucine, serine, proline amino acids, as well as TSS content, and TAA activity, showed a significantly enhancing trend in regenerative treatments compared to their corresponding non-regenerative selective treatments. The highest accumulated levels of arginine (43.27 μmol/g), lysine (2.56 μmol/g), methionine (5.43 μmol/g), glutamine (35.41 μmol/g), histidine (1.45 μmol/g), threonine (5.3 μmol/g), leucine (5.17 μmol/g), serine (3.93 μmol/g), proline (18.19 μmol/g), and TSS (96.5 mg g^−1^ DW) were detected in the PR42 treatment which displayed the highest regeneration rate as well as the highest mean number of regenerated shoot/explant (Supplementary data’ Table S4). The same trend of metabolite alterations was also detected in the other regenerative treatments compared to the control and selected non-regenerative treatments. On the contrary, non-regenerative treatments contained significantly higher amounts of TPC and MDA compared to the regenerative treatments, which indicated serious damage to the explants under inappropriate conditions of these treatments (Fig. 4).

## Discussion

Establishing high-performance indirect regeneration protocols has become the most challenging portion of novel plant breeding programs, as every plant species or genotype needs a specific regeneration protocol, especially recalcitrant woody species.

In the current study, the interaction of TDZ × IBA had successfully stimulated callogenesis and morphogenic calli development up to 13% at best, which showed a relatively low morphogenic rate efficiency. However, compared to the control treatment, a cross-talk between auxin and cytokinin is crucial for CI and MCI since the treatments without TDZ or IBA in the medium were not morphogenic, although some had produced callus. Previous studies have well documented that TDZ has effectively stimulated de novo morphogenesis in many recalcitrant plant species, such as *Phoenix dactylifera* [18], *Cocos nuciera* [19], etc. The cross-talk of TDZ × IBA could strongly trigger cell proliferation and de novo morphogenesis in many plant species including in vitro recalcitrants, because TDZ act as a very stable CK-like substance in plant systems that effectively participates in CK metabolism and auxin translocation and accumulation in the plant [20], on the other hand, IBA is also a well-known potent auxin for cell differentiation and proliferation and is widely used in callogenesis, morphogenic callus induction, and rooting events in most plant species, including the *Ficus* genus [4, 7], as observed in the current study in *F. lyrata*. The increased calli mass and formation of morphogenic calli in CI and MCI phases due to the presence of auxin and CK in the medium led to the promotion of new meristematic primordia and prevented their differentiation into roots [9]. In the case of Auxin’s role in stimulating morphogenic calli, [21] reported that the auxin-mediated induction of the *LBD29* gene in *Arabidopsis* promotes an array of other genes that stimulate callus induction, especially those responsible for ROS scavenging, lipid metabolism, cell wall hydrolysis, and methylation.

Shoot regeneration from the morphogenic calli occurred in the third week after the explants were cultured on the PR media. Like the MCI phase, shoot regeneration from morphogenic calli in the PR phase had occurred in a mid-range concentration of NO and a low concentration of auxin (NAA), but a high concentration of both types of adenine-based (BAP) and phenyl urea (TDZ) cytokinins were needed. This is also reported in many studies as a potent treatment for de novo organogenesis stimulation, especially for recalcitrant species [22–24]. The stimulatory effect of high CKs concentrations was reported to over-express *WUS* genes that encoded the major meristem forming regulators and the expression of many different downstream genes related to shoot development [25]. Moreover, the accumulation of CKs in cells causes the over-activation of the CK-oxidase enzyme which in turn leads to the generation of a large amount of adenine as the precursor for natural CK biosynthesis and stimulates the formation of new meristematic centers and shoot development [7].

Our findings here on *F. lyrata*, together with findings of several other studies in various plant species have proved that synergistic interaction of NO × CKs × auxin could strongly promote callogenesis, morphogenic callus induction, and shoot regeneration. This was supported by the reports in *Malus hupehensis* [26], *Albizzia lebbeck* [27], *Vanilla planifolia* [28], Cherry rootstocks [29, 30], *Malus niedzwetzkyana* [31], etc. The synergistic cross-talk of NO × CK × auxin dramatically stimulated cell dedifferentiation and cell proliferation in *Arabidopsis* [32] by augmenting the auxin and CK signaling pathways, and resulted in new meristematic centers. The synergistic contribution of auxins and NO in *Arabidopsis* remarkably activate the mitogen-activated protein kinase (*MAPK*) signaling and the *TIR*, an auxin receptor [33], as well as upregulating suppression of IAA oxidase activity, which cause enhancing the endogenous auxin accumulation in cells [34]. NO also fortifies the enzymatic and non-enzymatic cell defensive systems which cause amelioration of oxidative stresses and cell membrane integrity maintenance during the formation of meristematic centers [34, 35]. In a study on de novo morphogenesis of *Arabidopsis* using a NO-deficit mutant genotype, [36] it was discovered that NO and CK have a synergistic cross-talk in stimulating cell re-differentiation, cell division, and shoot regeneration by promoting the expression of a cell cycle gene *CYCD3;1* as a determinant factor.

Based on our findings in the current study, the PGRs interactions could switch the explants’ cell fate to the morphogenic and regenerative phase specifically through changes induced in the biosynthetic pathways and cell physiological responses. The morphogenic and regenerative treatments may have changed the cell's epigenetic reprogramming to the biosynthesis of essential cell metabolites necessary for stimulating cell redifferentiation and the indirect regeneration process. In this respect, [37] a significant difference between the metabolic contents of non-embryogenic callus (NEC), embryogenic callus (EC), and somatic embryos (SE) explants of the *Silybum marianum* plant has been reported. Accordingly, NEC explants contained a higher TSS content, probably showing stressful condition compared to EC and SE explants. On the contrary, EC and SE explants had accumulated significantly greater amounts of total free amino acids, especially proline and glutamine, which were crucial to developing these steps.

Soluble sugars are another group of cell metabolites that effectively contribute to de novo morphogenesis, mainly through supporting cell division and cell modifications via energy metabolism, keeping cell turgor and cell membrane integrity [38]. The over-accumulated TSS in the control and non-morphogenic treatments in the CI phase could be due to the absorbed sucrose from the medium that was not used in cell biological pathways because of the lack of cell division. Gautier *et. al*., [38] reported that the embryonal mass explants of Douglas-fir significantly accumulated greater contents of total carbohydrates compared to non-embryogenic calli, and of all carbohydrates, glucose, sucrose, and fructose displayed the greatest differences.

Phenolic compounds have essential roles in many cell biological events, considering their antioxidant potential [39], lignin biosynthesis, regulation of auxin transport, etc. [40]. But, their over-secretion by explants could be disastrous when polymerized by polyphenol oxidase enzyme preventing nutritional absorption from the medium, stopping cell division, and finally, death of the explant [4], as observed in non-morphogenic and non-regenerative treatments in the current study.

Compared to the morphogenic treatments, the remarkably increased TAA activity of the control and non-morphogenic treatments in the CI phase could be a reflection of their response to the induced-stress conditions resulting from the inappropriate PGRs × NO treatments. However, after the subculture to the MCI phase, their TAA activity drastically decreased due to prolonged exposure to non-morphogenic treatment and serious damage to the explants tissue. On the other hand, the significantly higher TAA activity of morphogenic and regenerative treatments might be due to ROS metabolism during rapid and continuous cell proliferation and shoot development. This event has been also reported in de novo morphogenesis and somatic embryogenesis in other studies on strawberry [41], and *Artemisia absinthium* [39].

Amino acids also effectively promote the de novo morphogenesis procedure, and therefore in many studies, some amino acids and organic additives such as casein hydrolysate (which contains 18 amino acids) alone or in combination are added to the medium to overcome recalcitrancy [42]. Kawade et al*.* [43]*,* have reported that arginine contributed to integrating metabolic and de novo morphogenesis processes of *Physcomitrium paten*, specifically through regulating the expression of *PpAN3* and *AtAN3* genes, which are involved in promoting cell proliferation and post-mitotic cell expansion. Furthermore, arginine is the main precursor for polyamines biosynthesis, and polyamines play a crucial role in promoting de novo plant morphogenesis [44]. Amino acids asparagine and glutamine are key metabolites for nitrogen and carbon storage and transport in plant systems, and more importantly, glutamine is involved in plant antioxidant defensive system, and asparagine is a precursor for the biosynthesis of some other essential amino acids e.g., lysine, threonine, methionine, and leucine, as well as nitrogenous compounds such as nucleotides, phytohormones, and secondary metabolites [45]. Thus, they are essentially involved in stimulating the de novo morphogenesis procedure, which is in close agreement with our findings. Mamedes-Rodrigues et al., [46] have revealed that amino acids are key determinant molecules to acquire embryogenic competency in *Brachypodium distachyon*, and their changing patterns can be considered as embryogenesis biomarkers. In another study, [47] also reported that glutamine and asparagine significantly improved shoot induction, elongation rate, and transformation efficiency of soybean plants. Glycine and proline also significantly increased in the MCI and PR phases in the current study, which indicate their important role in the indirect regeneration of *F. lyrata*. In agreement with our results, adding 500 mg/l of proline and 500 mg/l glutamine to the MS medium either alone or in combination remarkably enhanced callogenesis and shoot regeneration of rice explants [48]. Ramakrishnan et al., [49] achieved the highest rates of callogenesis and embryogenesis in *Allium cepa* shoot tip explants by enriching the culture medium with glycine, proline, and casein hydrolysate.

## Conclusion

In the current study, a high-efficient three-phase indirect regeneration protocol was developed in *Ficus lyrata* using leaf explants, MT medium, and different auxin × cytokinins × nitric oxide interactions. As *F. lyrata* is a recalcitrant woody species for indirect regeneration, we used nitric oxide to overcome recalcitrancy based on a synergistic interaction with auxin and cytokinins. The metabolites also were evaluated in each phase to discover the alteration trend and the main players. Results revealed that a synergistic interaction of a mid-range nitric oxide × high cytokinin × low auxin concentrations gave rise to the maximum morphogenic calli formation and shot regeneration. The results also revealed that nitric oxide had a key role in increasing morphogenic calli induction and a vital role in shoot regeneration. The morphogenic and regenerative treatments led to the similar metabolite alterations including the significant increase in biosynthesis of arginine, lysine, methionine, asparagine, glutamine, histidine, threonine, leucine, glycine, serine amino acids, TSS content, and TAA activity. We concluded that the proper PGR interactions in the MCI and PR phases could alter the biosynthesis pathways which led to the promotion of cell de-differentiation, cell proliferation, organogenic center formation, and shoot regeneration.

## Materials and methods

### Plant material and leaf explants preparation

The *F. lyrata* mother plants used in this study with voucher number 532 were identified by a botanist (Dr. Kazem Negaresh), and voucher specimens were deposited in the herbarium of the Botanical Laboratory of Ornamental Plant Research Institute at the Agricultural Sciences and Natural Resources University of Khuzestan, Iran. The routine maintenance of the mother plants was in accordance to the scientific instructions of the International Union for the Protection of Plant Varieties (UPOV).

For explant preparation, the current seasons’ fully expanded leaves from one-year *F. lyrata* plants were collected from the greenhouse and transferred to the plant tissue culture laboratory of the Department of Horticultural Science, at Agricultural Sciences and Natural Resources University of Khuzestan. For initiation, leaves were thoroughly washed and then they were divided into approximately 3 × 3 cm segments. Surface sterilization was done by immersion of leaf segments in 70% ethanol for 30 s, followed by sinking in 10% commercial bleach solution (containing 5% active chlorine) for 15 min, and finally, three times washing with sterilized distilled water under a laminar airflow cabinet.

Initiation success and survival of the explants were evaluated using the NO (0, 20, 30 & 40 uM) and TDZ (0, 4.5, 9,0 uM) combinations. At the end of the fourth week, the survival rate for each treatment were calculated using the following formula:

$$\mathrm{Survival\ rate }\left(\mathrm{\%}\right)= \left.\left(\frac{\mathrm{Number\ of\ explants\ survived}}{\mathrm{Total\ number\ of\ explants\ initiated}}\right.\right) \times 100$$

### Callus induction phase (CI phase)

For callus induction, the sterilized leaf samples (approximately 2 × 1.5 cm) were cultured into 250 ml jars each containing 30 ml of MT medium [50] enriched with 87.64 mM sucrose, 41.6 mM activated charcoal, 19.32 mM agar, and different combinations of TDZ (0 μM, 2.27 μM, 4.54 μM, 9.08 μM) × IBA (0 μM, 2.4 μM, 4.9 μM, 9.8 μM) × sodium nitroprusside (NO donor) (0 μM, 10 μM, 20 μM, 40 μM) as displayed in Table 1. The media's pH was adjusted to 5.9 and sterilized by autoclaving at 121 °C for 20 min. After culturing, the jars were placed in a growth chamber at 27 ± 2 °C in darkness and relative humidity of 60% for four weeks. At the end of the fourth week, the callus induction percentage for each treatment were calculated using the following formula:

$$\mathrm{Callus\ induction }\left(\mathrm{\%}\right)= \left.\left(\frac{\mathrm{Number\ of\ explants\ that}\ \mathrm{produced\ callus}}{\mathrm{Total\ number\ of\ initiated\ explants}}\right.\right) \times 100$$

### Morphogenic callus induction phase (MCI phase)

For morphogenic callus induction, all explants were subcultured to a fresh but the same media as the CI phase and placed under a condition of 25 ± 2 °C, light intensity of 60 μmol m^−2^ s^−1^, and relative humidity of 60% for another four weeks. In the end, the explants were screened for browning index (visually) and morphogenic callus induction. Two types of calli were distinguished as follows: A: Non-morphogenic calli with white-creamy to brownish in color and a friable or watery texture (Fig. 1D, E), and B: morphogenic calli with a green to dark green color, compact texture, and a granulate shape (Fig. 1F). The morphogenic callus induction (%) for each treatment was estimated by the following formula:

$$\mathrm{Morphogenic\ callus\ induction }\left(\mathrm{\%}\right)= \left.\left(\frac{\mathrm{Total\ number\ of\ explants\ with\ morphogenic\ callus}}{\mathrm{Total\ number\ of\ incubated\ explants}}\right.\right) \times 100$$

### Plant regeneration (PR phase)

For plant regeneration, morphogenic calli from the MCI phase were subcultured on PR media at the end of the eighth week. The explants were first slightly trimmed by cutting a thin layer of the lower parts (to increase the contact surface area of explants with the media), and cultured on MT medium enriched with 87.64 mM sucrose, 41.6 mM activated charcoal, 20.21 mM agar supplemented with different concentrations of BAP × TDZ × sodium nitroprusside (NO donor) as presented in Table 2.

The cultures were then placed in a growth chamber under the condition of 16/8 h (light/darkness) photoperiod (light intensity of 60 μmol m^−2^ s^−1^), relative humidity of 60%, and 23 ± 1 °C for four weeks. The regeneration percentage and the mean number of shoots per explant for each treatment were recorded using the following formula, and shoot length was measured directly by a ruler.

$$\mathrm{Regeneration\ percentage }= \left.\left(\frac{\mathrm{Total\ number\ of\ explants\ with\ regenerated\ shoots}}{\mathrm{Total\ number\ of\ incubated\ calli}}\right.\right) \times 100$$

$$\mathrm{Mean\ number\ of\ regenerated\ shoots\ per\ explant }= \left.\left(\frac{\mathrm{Total\ number\ of\ regenerated\ shoot}/explant}{\mathrm{Total\ number\ of\ incubated\ explants}}\right.\right) \times 100$$

### Metabolite profiles

Metabolite profiles of explants in CI, MCI, and PR phases were analyzed for eight selected treatments that included the morphogenic and non-morphogenic treatments in the CI and MCI phases as well as regenerative and non-regenerative treatments in the PR phase alongside the control treatments for each phase (Supplementary data, Table S4). The sampling from the explants was performed in the third week after subculturing at each phase. Accordingly, one gram sample for all treatments was prepared, then the samples were immediately ground to fine powder in liquid nitrogen using a mortar and pestle, and finally, they were lyophilized and stored at -80 °C for further investigations.

### Amino acid extraction and analysis

The free amino acid content of samples was evaluated according to [51] method. The extraction was done by mixing 50 mg of stored powder with 1 ml of 0.01 M hydrochloric acid (HCl) and vortexing for 1 min, then the mixture left to stand for 1 h. at room temperature. The samples were then centrifuged at 14,000 g for 5 min and 1 ml supernatant was transferred into the new glass tubes after filtering by 0.22 μm syringe filters. The derivation phase was performed by adding 5 μl of pyridine and 5 μl of methyl chloroformate to the mixtures and shaken for 5 min. The procedure continued by adding 90 μl of 50 mM sodium bicarbonate to the mixtures and leaving them to stand for 5 min, then centrifuged at 10,000 g for 3 min. Following that, 50 μl of the lower phase was transferred to a new glass tube containing a few crystals of anhydrous sodium sulfate, left to stand for 30 min, and finally, 50 μl samples were transferred to the new tubes and used for UPLC/MS analysis. The free amino acid content of samples was determined using an ACQUITY UPLC H-Class System device equipped with an ACQUITY QDa Mass Detector (Waters Corporation, Milford, MA, USA), with an analytical column C18 (2.1 × 50 mm, 1.7 μm; Waters Corporation). The flow rate was 500 μl/min with an injection volume of 5 μl, and the chromatography mobile phase consisted of an isocratic mixture of 30% (v/ v) HPLC-grade water and 70% (v/v) acetonitrile + 0.1%formic acid. Various concentrations of standard solutions (0.02 μg/ml to 200 μg/ ml) for all amino acids were prepared and used for plotting the standard curves.

### Total phenolic content (TPC) assay

According to [52], total phenolic content of all samples was determined using Folin-Ciocalteu method. Accordingly, 2 ml of extraction buffer containing methanol/distilled water /HCl (8:1.9:0.1 v/v/v) was poured on 200 mg of stored powder and kept at room temperature for 2 h and then centrifuged for 15 min at 7000 g. Following that, a reagent solution was made by mixing 100 μL of supernatant and 750 μL of ten-times pre-diluted Folin-Ciocalteu and left to stand for five min at room temperature, then 750 μL of 0.7 M sodium bicarbonate solution was added to the mixture and allowed to stand for 90 min. Finally, the total phenolic content of each sample was recorded using a microplate spectrophotometer (Epoch, BioTek, USA) and absorbance at 725 nm.

### Total antioxidant activity (TAA) assay

TAA was determined using [53] method by adding 10 ml of methanol/distilled water (8:2 v: v) solution to the 200 mg of stored powder and keeping it at room temperature for 30 min, after shaking well. The mixture was centrifuged at 7000 g for 10 min, then 100 μL of supernatant was added to 900 μL of 0.1 mM DPPH reagent and 1 ml Tris–HCl (pH = 7.5) buffer, vortexed and kept at room temperature in dark for 30 min. The TAA activity of each sample was recorded using a microplate spectrophotometer (Epoch, BioTek, USA) at 725 nm and using the following formula:

$$\mathrm{TAA }\left(\mathrm{\%}\right)= \left.\left(1- \frac{\mathrm{Absorbance\ of\ sample}}{\mathrm{Absorbance\ of\ control}}\right.\right) \times 100$$

### Total soluble sugars (TSS) assay

According to [54], to determine the TSS content of samples, 10 ml of ethanol/distill water (8:2 v/v) solution was added to 100 mg of stored powder and kept for 30 min under room temperature then centrifuged at 10,000 g for 15 min at 4 °C. Following that, 1 ml of supernatant was added to 3 ml of Anthrone reagent (150 mg Anthrone + 100 ml H_2_SO_4,_ 72%) and heated at 100 °C in a water bath for 15 min, then immediately cooled by placing on ice. The TSS content of samples was recorded using a microplate spectrophotometer (Epoch, BioTek, USA) and absorbance at 620 nm.

### Malondialdehyde content (MDA)

MDA content was assessed to determine the lipid peroxidation state of samples. According to the [55] method, 100 mg of fresh leaf samples were ground to fine powder in liquid nitrogen and mixed with 2 ml ethanol: distilled water (80% v/v), and centrifuged at 10,000 g for 15 min. Afterward, 1 ml of supernatant was taken and added to 1 ml thiobarbituric acid (TBA) solution consisting of 20.0% (w/v) TCA and 0.01% butylated hydroxytoluene. The samples were then heated at 95 °C for 30 min and instantly cooled on ice, then centrifuged at 7000 g for another 15 min. The MDA content of samples was measured using a spectrophotometer (Epoch, BioTek, United States) at 414, 532, and 600 nm and content was estimated using the following formula:

$$\mathrm{MDA}= 6.45 \left(\mathrm{Abs }\ 532-\mathrm{Abs }\ 600\right)-0.56\ \mathrm{ Abs }\ 414$$

### Elongation, rooting and acclimatization

The regenerated shootlets (0.5–2 cm in length) were cultured on MS medium [56] supplemented with 1.5 μM BAP + 0.5 μM GA3 and the condition of 16/8 h (light/darkness) photoperiod (light intensity of 60 μmol m^−2^ s^−1^), relative humidity of 60%, and 27 ± 1 °C, for elongation and preparing for transferring to the rooting stage. After three weeks, shoots with at least 3 cm in length were cultured on the rooting medium consisting of half-strength MS medium, 58.42 mM sucrose, 83.2 mM activated charcoal, 1.23 μM IBA, and 16.81 mM agar and kept for four weeks under the condition of 12/12 h (light/darkness) photoperiod (light intensity of 40 μmol m^−2^ s^−1^), relative humidity of 60%, and 27 ± 1 °C to develop the roots. Then, for acclimatization, the plantlets were transferred to a greenhouse and cultured into plastic trays containing coconut peat and fine perlite in a 7: 3 ratio and kept under a 25/18 ± 2 °C Day/night temperature, 85% relative humidity, and light intensity of 60 μmol m^−2^ s^−1^ conditions for four weeks.

### Experimental design and statistical analysis

The experiment was carried out in three phases and each phase was conducted in a factorial experiment with three factors (NO × TDZ × IBA in the CI and MCI phases, and NO × BAP × TDZ in the PR phase) in a completely randomized design with 3 replications for each treatment and 5 explants per replicate. Data for CI, MCI, and PR stages were subjected to 3-way ANOVA. Data for metabolites (amino acids, TSS, TPC, TAA, and MDA) were collected from selected treatments and subjected to one-way ANOVA. All data were analyzed by SAS software version 9.4 (SAS institute, USA), and the means were compared by Duncan's multiple range test (*p* ≤ 0.05).

## Supplementary Information

**Additional file 1:** **Table S1. **The 3-way ANOVA for the effect of NO, TDZ, andIBA and their interactions on survival rate (%),browning index, callusinduction (%), and morphogenic callus induction (%) parameters in *F.lyrata* leaf explants cultured on MT medium. **TableS2.** The 3-way ANOVA forthe effect of BAP, TDZ, and NO and their interactions on regeneration (%),shoot/explant, and shoot length parameters in *F. lyrata* explantscultured on MT medium. **Table S3.** Effect of different auxin × cytokinins × nitricoxide interactions on shoot regeneration (%), number of shoots/explant, andshoot length parameters of *F. lyrata* explants cultured on MT medium. **TableS4.** Effect of differentauxin × cytokinin × nitric oxide interactions (selective treatments) onphytochemical properties of F. lyrata explants in different indirect de novoregeneration phases, cultured on MT medium.

**Additional file 2.** 

**Additional file 3.** 

## Data Availability

The datasets during and/or analysed during the current study available from the corresponding author on reasonable request.

## Abbreviations

BAP: 6-Benzyl amino purine
CI: Callus induction
CK: Cytokinin
GA3: Gibberellic acid
IBA: Indole-3-butyric acid
MCI: Morphogenic callus induction
NAA: 1-Naphthalene acetic acid
NO: Nitric oxide
PGR: Plant growth regulator
PR: Plant regeneration
TDZ: Thidiazuron
