# Supplementary material for: Indirect regeneration in Ficus lyrata Warb. and metabolite profiles influenced by nitric oxide and Plant growth regulators
Source: BMC Plant Biol. 2023 Jun 17;23:325. doi: 10.1186/s12870-023-04339-z (PMC10276444; doi:10.1186/s12870-023-04339-z)
Supplement: Supplementary file 1 — Additional file 1: Table S1. The 3-way ANOVA for the effect of NO, TDZ, andIBA and their interactions on survival rate (%),browning index, callusinduction (%), and morphogenic callus induction (%) parameters in F.lyrata leaf explants cultured on MT medium. TableS2. The 3-way ANOVA forthe effect of BAP, TDZ, and NO and their interactions on regeneration (%),shoot/explant, and shoot length parameters in F. lyrata explantscultured on MT medium. Table S3. Effect of different auxin × cytokinins × nitricoxide interactions on shoot regeneration (%), number of shoots/explant, andshoot length parameters of F. lyrata explants cultured on MT medium. TableS4. Effect of differentauxin × cytokinin × nitric oxide interactions (selective treatments) onphytochemical properties of F. lyrata explants in different indirect de novoregeneration phases, cultured on MT medium. [file 12870_2023_4339_MOESM1_ESM.docx]

| **Table S1** The 3-way ANOVA for the effect of NO, TDZ, and IBA and their interactions on survival rate (%), browning index, callus induction (%), and morphogenic callus induction (%) parameters in *F. lyrata* leaf explants cultured on MT medium. | | | | | |
| --- | --- | --- | --- | --- | --- |
| **Source of variance** | **DF** | **Survival rate (%)** | **Browning index** | **Callus induction (%)** | **Morphogenic callus**  **induction (%)** |
|  |  | **Mean Square** | **Mean Square** | **Mean Square** | **Mean Square** |
| NO | 3 | 7880.32407****** | 27.43518519****** | 1765.74074****** | 3707.40741****** |
| **TDZ** | 2 | 28663.19444****** | 22.96527778****** | 23275.00000****** | 3411.11111****** |
| **IBA** | 3 | 1124.76852****** | 1.43518519****** | 24943.51852****** | 3396.29630****** |
| **NO * TDZ** | 6 | 4270.60185****** | 3.40046296****** | 3849.07407****** | 1285.18519****** |
| **NO * IBA** | 9 | 249.45988 ^ns^ | 1.36111111****** | 943.51852****** | 1630.86420****** |
| **TDZ * IBA** | 6 | 192.82407 ^ns^ | 1.20601852****** | 2371.29630****** | 962.96296****** |
| **NO * TDZ * IBA** | 18 | 232.33025 ^ns^ | 1.67824074****** | 945.37037****** | 708.64198****** |
| **CV** |  | 10.68 | 17.54 | 16.49 | 18.22 |
| ** Significance and ^ns^ Non significance at the 5% level probability. | | | | | |

**Supplementary data**

| **Table S2** The 3-way ANOVA for the effect of BAP, TDZ, and NO and their interactions on regeneration (%), shoot/explant, and shoot length parameters in *F. lyrata* explants cultured on MT medium. | | | | |
| --- | --- | --- | --- | --- |
| **Source of variance** | **DF** | **Regeneration (%)** | **Shoot/explant** | **Shoot length (cm)** |
|  |  | **Mean Square** | **Mean Square** | **Mean Square** |
| BAP | 2 | 4669.44444****** | 38.2439174****** | 0.95550625****** |
| **TDZ** | 3 | 1558.33333****** | 13.1238062****** | 0.31874699****** |
| **NO** | 3 | 1780.55556****** | 15.6299285****** | 0.32250625****** |
| **BAP * TDZ** | 6 | 1558.33333****** | 13.1238063****** | 0.31874699****** |
| **BAP * NO** | 6 | 1780.55556****** | 15.6299285****** | 0.32250625****** |
| **TDZ * NO** | 9 | 595.37037****** | 6.2643359****** | 0.10777662****** |
| **BAP * TDZ * NO** | 18 | 595.37037****** | 6.2643359****** | 0.10777662****** |
| **CV** |  | 18.51 | 14.97 | 14.42 |
| ** Significance and ^ns^ Non significance at the 5% level probability. | | | | |

| **Table S3** Effect of different auxin × cytokinins × nitric oxide interactions on shoot regeneration (%), number of shoots/explant, and shoot length parameters of *F. lyrata* explants cultured on MT medium. | | | | | | | |
| --- | --- | --- | --- | --- | --- | --- | --- |
| BAP (µM) | TDZ (µM) | Nitric oxide (µM) | NAA (µM) | Treatment code | Regeneration (%) | Shoot/explant | Shoot length (cm) |
| 0 | 0 | 0 | 0.53 | PR0 | 0 ± 0 d | 0 ± 0 e | 0 ± 0 d |
|  |  | 10 | 0.53 | PR1 | 0 ± 0 d | 0 ± 0 e | 0 ± 0 d |
|  |  | 20 | 0.53 | PR2 | 0 ± 0 d | 0 ± 0 e | 0 ± 0 d |
|  |  | 40 | 0.53 | PR3 | 0 ± 0 d | 0 ± 0 e | 0 ± 0 d |
|  | 1.14 | 0 | 0.53 | PR4 | 0 ± 0 d | 0 ± 0 e | 0 ± 0 d |
|  |  | 10 | 0.53 | PR5 | 0 ± 0 d | 0 ± 0 e | 0 ± 0 d |
|  |  | 20 | 0.53 | PR6 | 0 ± 0 d | 0 ± 0 e | 0 ± 0 d |
|  |  | 40 | 0.53 | PR7 | 0 ± 0 d | 0 ± 0 e | 0 ± 0 d |
|  | 2.25 | 0 | 0.53 | PR8 | 0 ± 0 d | 0 ± 0 e | 0 ± 0 d |
|  |  | 10 | 0.53 | PR9 | 0 ± 0 d | 0 ± 0 e | 0 ± 0 d |
|  |  | 20 | 0.53 | PR10 | 0 ± 0 d | 0 ± 0 e | 0 ± 0 d |
|  |  | 40 | 0.53 | PR11 | 0 ± 0 d | 0 ± 0 e | 0 ± 0 d |
|  | 4.5 | 0 | 0.53 | PR12 | 0 ± 0 d | 0 ± 0 e | 0 ± 0 d |
|  |  | 10 | 0.53 | PR13 | 0 ± 0 d | 0 ± 0 e | 0 ± 0 d |
|  |  | 20 | 0.53 | PR14 | 0 ± 0 d | 0 ± 0 e | 0 ± 0 d |
|  |  | 40 | 0.53 | PR15 | 0 ± 0 d | 0 ± 0 e | 0 ± 0 d |
| 8.9 | 0 | 0 | 0.53 | PR16 | 0 ± 0 d | 0 ± 0 e | 0 ± 0 d |
|  |  | 10 | 0.53 | PR17 | 0 ± 0 d | 0 ± 0 e | 0 ± 0 d |
|  |  | 20 | 0.53 | PR18 | 0 ± 0 d | 0 ± 0 e | 0 ± 0 d |
|  |  | 40 | 0.53 | PR19 | 0 ± 0 d | 0 ± 0 e | 0 ± 0 d |
|  | 1.14 | 0 | 0.53 | PR20 | 0 ± 0 d | 0 ± 0 e | 0 ± 0 d |
|  |  | 10 | 0.53 | PR21 | 0 ± 0 d | 0 ± 0 e | 0 ± 0 d |
|  |  | 20 | 0.53 | PR22 | 0 ± 0 d | 0 ± 0 e | 0 ± 0 d |
|  |  | 40 | 0.53 | PR23 | 0 ± 0 d | 0 ± 0 e | 0 ± 0 d |
|  | 2.25 | 0 | 0.53 | PR24 | 0 ± 0 d | 0 ± 0 e | 0 ± 0 d |
|  |  | 10 | 0.53 | PR25 | 0 ± 0 d | 0 ± 0 e | 0 ± 0 d |
|  |  | 20 | 0.53 | PR26 | 0 ± 0 d | 0 ± 0 e | 0 ± 0 d |
|  |  | 40 | 0.53 | PR27 | 0 ± 0 d | 0 ± 0 e | 0 ± 0 d |
|  | 4.5 | 0 | 0.53 | PR28 | 0 ± 0 d | 0 ± 0 e | 0 ± 0 d |
|  |  | 10 | 0.53 | PR29 | 0 ± 0 d | 0 ± 0 e | 0 ± 0 d |
|  |  | 20 | 0.53 | PR30 | 0 ± 0 d | 0 ± 0 e | 0 ± 0 d |
|  |  | 40 | 0.53 | PR31 | 0 ± 0 d | 0 ± 0 e | 0 ± 0 d |
| 17.8 | 0 | 0 | 0.53 | PR32 | 0 ± 0 d | 0 ± 0 e | 0 ± 0 d |
|  |  | 10 | 0.53 | PR33 | 0 ± 0 d | 0 ± 0 e | 0 ± 0 d |
|  |  | 20 | 0.53 | PR34 | 0 ± 0 d | 0 ± 0 e | 0 ± 0 d |
|  |  | 40 | 0.53 | PR35 | 0 ± 0 d | 0 ± 0 e | 0 ± 0 d |
|  | 1.14 | 0 | 0.53 | PR36 | 0 ± 0 d | 0 ± 0 e | 0 ± 0 d |
|  |  | 10 | 0.53 | PR37 | 0 ± 0 d | 0 ± 0 e | 0 ± 0 d |
|  |  | 20 | 0.53 | PR38 | 0 ± 0 d | 0 ± 0 e | 0 ± 0 d |
|  |  | 40 | 0.53 | PR39 | 0 ± 0 d | 0 ± 0 e | 0 ± 0 d |
|  | 2.25 | 0 | 0.53 | PR40 | 0 ± 0 d | 0 ± 0 e | 0 ± 0 d |
|  |  | 10 | 0.53 | PR41 | 46.66 ± 4.66 c | 3.41 ± 0.93 d | 0.86 ± 0.12 c |
|  |  | 20 | 0.53 | PR42 | 86.66 ± 6.33 a | 10.46 ± 2.11 a | 1.07 ± 0.13 a |
|  |  | 40 | 0.53 | PR43 | 0 ± 0 d | 0 ± 0 e | 0 ± 0 d |
|  | 4.5 | 0 | 0.53 | PR44 | 0 ± 0 d | 0 ± 0 e | 0 ± 0 d |
|  |  | 10 | 0.53 | PR45 | 53.33 ± 8.46 b | 4.8 ± 1.33 c | 0.94 ± 0.09 b |
|  |  | 20 | 0.53 | PR46 | 86.66 ± 6.13 a | 6.06 ± 1.66 b | 1.03 ± 0.14 a |
|  |  | 40 | 0.53 | PR47 | 0 ± 0 d | 0 ± 0 e | 0 ± 0 d |
| Means in each column with the same letters are not significantly different at p < 0.05, according to Duncan's multiple range tests. Each treatment includes three replicates, and each replicate contains five explants. Each value represents the mean ± SE. | | | | | | | |

| **Table S4** Effect of different auxin × cytokinin × nitric oxide interactions (selective treatments) on phytochemical properties of F. lyrata explants in different indirect de novo regeneration phases, cultured on MT medium. | | | | | | | | | | | | | | | | | |
| --- | --- | --- | --- | --- | --- | --- | --- | --- | --- | --- | --- | --- | --- | --- | --- | --- | --- |
| Treatment code | | | Callus induction phase (CI) | | | | | | | | | | | | | | |
|  |  |  | **Arginine**  **(μmol/g)** | **Lysine**  **(μmol/g)** | **Methionine**  **(μmol/g)** | **Asparagine**  **(μmol/g)** | **Glutamine**  **(μmol/g)** | **Histidine**  **(μmol/g)** | **Threonine**  **(μmol/g)** | **Glycine**  **(μmol/g)** | **Leucine**  **(μmol/g)** | **Serine**  **(μmol/g)** | **Proline**  **(μmol/g)** | **TPC**  **(mg GA E/g DW)** | **TSS (mg g^-1^ DW)** | **TAA (%)** | **MDA (mmol g^-1^ FW)** |
| CI 0 | | | 3.43 ± 0.35 g | 0.29 ± 0.06 e | 0.07 ± 0.02 h | 5.48 ± 0.57 e | 2.35 ± 0.2 e | 0.05 ± 0.01 e | 0.2 ± 0.08 e | 14.62 ± 0.63 a | 6.79 ± 0.2 cd | 3.24 ± 0.12 b | 17.13 ± 0.37 b | 6.71 ± 0.08 a | 68.03 ± 0.55 a | 79.34 ± 0.66 a | 1.43 ± 0.05 a |
| CI 6 | | | 15.29 ± 0.46 d | 1.23 ± 0.02 cd | 8.35 ± 0.35 d | 24.02 ± 1.23 c | 19.38 ± 1.16 b | 1.19 ± 0.03 c | 4.36 ± 0.21 c | 7.35 ± 0.06 c | 6.33 ± 0.2 de | 1.88 ± 0.15 f | 7.81 ± 0.25 c | 3.24 ± 0.11 d | 57.9 ± 0.09 c | 77.7 ± 0.38 b | 0.55 ± 0.01 c |
| **CI 10** | | | 17.23 ± 0.22 c | 1.01 ± 0.03 d | 5.88 ± 0.06 e | 27.13 ± 1.02 c | 20.41 ± 0.66 ab | 1.78 ± 0.11 ab | 4.83 ± 0.08 c | 6.67 ± 0.06 c | 6.09 ± 0.08 e | 1.94 ± 0.06 ef | 8.18 ± 0.46 c | 3.13 ± 0.03 d | 51.16 ± 0.84 d | 75.16 ± 0.35 c | 0.53 ± 0.1 c |
| **CI 17** | | | 19.36 ± 0.25 b | 2.82 ± 0.07 a | 12.01 ± 0.53 c | 39.08 ± 1.16 b | 18.99 ± 0.66 b | 1.98 ± 0.1 a | 7.88 ± 0.24 ab | 6.49 ± 0.12 c | 7.72 ± 0.14 b | 2.17 ± 0.03 e | 5.02 ± 0.11 d | 2.83 ± 0.06 e | 62.76 ± 0.48 b | 63.3 ± 0.35 e | 0.15 ± 0.03 de |
| **CI 22** | | | 18.69 ± 0.45 b | 2.13 ± 0.04 b | 15.29 ± 0.19 a | 42.24 ± 1.44 b | 22.36 ± 0.52 a | 1.54 ± 0.15 b | 8.48 ± 0.35 a | 7.46 ± 0.11 c | 6.69 ± 0.08 cd | 2.81 ± 0.04 c | 5.01 ± 0.1 d | 2.55 ± 0.11 f | 69.13 ± 0.98 a | 61.96 ± 0.44 f | 0.27 ± 0.01 d |
| **CI 33** | | | 22.07 ± 0.4 a | 2.36 ± 0.22 b | 14.43 ± 0.13 b | 58.23 ± 1.7 a | 19.22 ± 0.81 b | 1.63 ± 0.13 b | 7.7 ± 0.25 b | 8.39 ± 0.38 c | 8.25 ± 0.17 a | 2.49 ± 0.03 d | 7.65 ± 0.12 c | 1.8 ± 0.09 g | 63.43 ± 0.43 b | 64.6 ± 0.59 d | 0.06 ± 0 e |
| **CI 39** | | | 8.89 ± 0.87 f | 1.35 ± 0.02 c | 2.76 ± 0.18 g | 12.49 ± 0.73 d | 6.08 ± 0.19 d | 1.02 ± 0.06 cd | 1.27 ± 0.08 d | 14.06 ± 1.8 a | 6.85 ± 0.15 cd | 3.73 ± 0.07 a | 18.12 ± 0.26 a | 5.26 ± 0.14 c | 68.76 ± 0.23 a | 78.03 ± 0.8 ab | 1.14 ± 0.06 b |
| **CI 46** | | | 13.64 ± 0.33 e | 1.52 ± 0.04 c | 3.59 ± 0.07 f | 7.78 ± 0.99 e | 9.56 ± 0.51 c | 0.83 ± 0.06 d | 1.43 ± 0.19 d | 11.51 ± 0.9 b | 6.89 ± 0.2 c | 2.85 ± 0.06 c | 18.2 ± 0.45 a | 5.62 ± 0.07 b | 61.33 ± 0.61 b | 74.86 ± 0.2 c | 1.34 ± 0.05 a |
|  | | **Morphogenic callus induction phase (MCI)** | | | | | | | | | | | | | | | |
| CI 0 | | | 0.92 ± 0.04 e | 0 ± 0 c | 0 ± 0 f | 1.28 ± 0.04 d | 3.16 ± 0.1 d | 0 ± 0 e | 0.07 ± 0.02 f | 4.38 ± 0.19 f | 0.06 ± 0.02 e | 0.45 ± 0.08 d | 4.63 ± 0.35 d | 7.36 ± 0.2 a | 52.17 ± 1.17 d | 47.19 ± 0.34 d | 2.47 ± 0.07 a |
| **CI 6** | | | 17.42 ± 0.49 d | 2.44 ± 0.2 ab | 6.39 ± 0.16 b | 16.89 ± 1.05 c | 24.8 ± 0.84 c | 1.25 ± 0.06 d | 3.2 ± 0.21 d | 18.69 ± 0.32 b | 4.32 ± 0.11 b | 1.84 ± 0.06 a | 8.68 ± 0.33 a | 4.14 ± 0.19 b | 72.46 ± 1.62 c | 75.94 ± 1.19 a | 0.82 ± 0.04 c |
| **CI 10** | | | 16.47 ± 1.19 d | 2.77 ± 0.16 a | 5.15 ± 0.14 c | 14.59 ± 0.32 c | 25.82 ± 1.25 c | 1.45 ± 0.13 d | 3.78 ± 0.22 c | 16.71 ± 0.25 d | 4.66 ± 0.19 b | 1.66 ± 0.04 ab | 8.37 ± 0.12 a | 3.93 ± 0.04 b | 76.55 ± 0.55 c | 77.26 ± 1.67 a | 0.74 ± 0.04 c |
| **CI 17** | | | 27.75 ± 0.66 b | 2.25 ± 0.05 b | 6.71 ± 0.18 b | 32.06 ± 1.85 b | 31.57 ± 1.69 b | 2.75 ± 0.15 c | 6.39 ± 0.22 b | 16.29 ± 0.11 d | 5.17 ± 0.15 a | 1.61 ± 0.04 ab | 5.6 ± 0.18 c | 3.73 ± 0.11 b | 92.61 ± 2.79 b | 72.89 ± 3.59 a | 0.07 ± 0 d |
| **CI 22** | | | 25.19 ± 0.35 c | 2.36 ± 0.2 ab | 8.26 ± 0.29 a | 28.98 ± 0.96 b | 29.58 ± 0.92 b | 4.85 ± 0.1 a | 6.31 ± 0.22 b | 17.78 ± 0.2 c | 5.5 ± 0.23 a | 1.73 ± 0.04 a | 5.28 ± 0.3 cd | 3.12 ± 0.04 e | 88.82 ± 2 b | 75.17 ± 1.95 a | 0.06 ± 0 d |
| **CI 33** | | | 36.88 ± 0.36 a | 2.32 ± 0.16 ab | 7.98 ± 0.09 a | 51.61 ± 2.09 a | 41.33 ± 1.24 a | 4.34 ± 0.12 b | 7.74 ± 0.23 a | 21.35 ± 0.45 a | 5.15 ± 0.1 a | 1.45 ± 0.13 b | 5.31 ± 0.09 cd | 1.26 ± 0.06 f | 114.49 ± 5.11 a | 77.31 ± 0.57 a | 0.02 ± 0 d |
| **CI 39** | | | 1.39 ± 0.24 e | 0.04 ± 0.01 c | 1.32 ± 0.08 d | 1.22 ± 0.1 d | 4.59 ± 0.21 d | 0 ± 0 e | 1.21 ± 0.12 e | 8.31 ± 0.34 e | 1.18 ± 0.09 d | 0.64 ± 0.1 cd | 7.98 ± 0.17 ab | 6.27 ± 0.15 c | 42.53 ± 0.45 e | 52.8 ± 0.5 c | 1.98 ± 0.04 b |
| **CI 46** | | | 1.91 ± 0.27 e | 0.02 ± 0 c | 0.82 ± 0.06 e | 2.03 ± 0.22 d | 3.8 ± 0.03 d | 0 ± 0 e | 1.52 ± 0.05 e | 9.08 ± 0.31 e | 1.8 ± 0.09 c | 0.73 ± 0.04 c | 7.51 ± 0.16 b | 6.82 ± 0.13 b | 54.82 ± 1.1 d | 60.48 ± 0.72 b | 2.07 ± 0.11 b |
|  | **Plant regeneration phase (PR)** | | | | | | | | | | | | | | | | |
| **PR0** | | | 1.46 ± 0.12 e | 0.05 ± 0.01 cd | 0.03 ± 0 d | 1.45 ± 0.09 e | 3.42 ± 0.21 d | 0.13 ± 0.04 c | 0 ± 0 c | 6.19 ± 0.23 de | 0.06 ± 0 e | 1.82 ± 0.12 c | 6.75 ± 0.11 c | 5.65 ± 0.14 c | 41.84 ± 2.62 e | 51.8 ± 1.92 e | 1.62 ± 0.05 ab |
| **PR14** | | | 0.96 ± 0.1 e | 0.12 ± 0.01 cd | 0.06 ± 0.02 d | 2.25 ± 0.23 e | 1.63 ± 0.04 de | 0.68 ± 0.15 b | 0 ± 0 c | 5.21 ± 0.19 ef | 0.5 ± 0.17 d | 1.16 ± 0.1 e | 4.25 ± 0.12 d | 6.42 ± 0.23 b | 56.21 ± 1.35 d | 46.04 ± 0.76 f | 1.46 ± 0.03 c |
| **PR26** | | | 2.01 ± 0.07 e | 0 ± 0cd | 0 ± 0 d | 1.06 ± 0.15 e | 1.15 ± 0.08 e | 0 ± 0 c | 0 ± 0 c | 4.59 ± 0.26 f | 0.18 ± 0.02 e | 1.54 ± 0.05 cd | 4.73 ± 0.13 d | 4.52 ± 0.2 d | 39.48 ± 1.66 e | 34.46 ± 1.31 h | 1.73 ± 0.06 a |
| PR30 | | | 2.65 ± 0.02 e | 0.26 ± 0.06 c | 0.07 ± 0.02 d | 2.23 ± 0.12 e | 2.3 ± 0.22 de | 0.08 ± 0.01 c | 0 ± 0 c | 6.83 ± 0.12 d | 0.92 ± 0.05 c | 1.37 ± 0.03 de | 5.16 ± 0.09 d | 7.23 ± 0.22 a | 53.46 ± 2.54 d | 41.27 ± 0.5 g | 1.54 ± 0.02 bc |
| **PR41** | | | 31.57 ± 1.22 d | 2.64 ± 0.15 a | 2.14 ± 0.1 c | 31.57 ± 1.96 d | 33.3 ± 1.13 b | 1.3 ± 0.13 a | 3.26 ± 0.25 b | 24.86 ± 0.55 a | 4.73 ± 0.1 b | 4.17 ± 0.03 a | 14.62 ± 0.1 b | 2.79 ± 0.3 e | 87.93 ± 1.89 b | 89.18 ± 1.24 a | 0.63 ± 0.03 d |
| **PR42** | | | 43.27 ± 0.88 a | 2.56 ± 0.08 a | 5.43 ± 0.46 a | 39.55 ± 0.16 b | 35.41 ± 0.54 a | 1.45 ± 0.17 a | 5.3 ± 0.27 a | 22.39 ± 0.56 b | 5.17 ± 0.14 a | 3.93 ± 0.2 ab | 18.19 ± 0.47 a | 2.45 ± 0.05 e | 96.5 ± 1.96 a | 71.75 ± 0.97 d | 0.23 ± 0.03 f |
| **PR45** | | | 34.84 ± 0.96 c | 2.26 ± 0.05 b | 5.79 ± 0.04 a | 34.33 ± 1.11 c | 31.34 ± 1.09 c | 1.37 ± 0.04 a | 5.27 ± 0.29 a | 20.52 ± 0.95 c | 5.36 ± 0.08 a | 4.2 ± 0.1 a | 13.74 ± 0.51 b | 2.61 ± 0.2 e | 75.53 ± 1.25 c | 77.33 ± 1.25 c | 0.44 ± 0.03 e |
| **PR46** | | | 37.77 ± 0.86 b | 2.22 ± 0.09 b | 4.22 ± 0.08 b | 42.18 ± 0.9 a | 35.59 ± 0.2 a | 1.63 ± 0.13 a | 4.86 ± 0.1 a | 24.83 ± 0.33 a | 4.52 ± 0.11 b | 3.74 ± 0.16 b | 14.63 ± 0.68 b | 2.17 ± 0.06 e | 80.13 ± 1.74 c | 81.39 ± 1.66 b | 0.58 ± 0.02 d |
| Means in each column with the same letters are not significantly different at p < 0.05, according to Duncan's multiple range tests. Each treatment includes three replicates, and each replicate contains five explants. Each value represents the mean ± SE. | | | | | | | | | | | | | | | | | |
